# Prediction of heme binding residues from protein sequences with integrative sequence profiles

**DOI:** 10.1186/1477-5956-10-S1-S20

**Published:** 2012-06-21

**Authors:** Yi Xiong, Juan Liu, Wen Zhang, Tao Zeng

**Affiliations:** 1 School of Computer, Wuhan University, Wuhan 430072, China

## Abstract

**Background:**

The heme-protein interactions are essential for various biological processes such as electron transfer, catalysis, signal transduction and the control of gene expression. The knowledge of heme binding residues can provide crucial clues to understand these activities and aid in functional annotation, however, insufficient work has been done on the research of heme binding residues from protein sequence information.

**Methods:**

We propose a sequence-based approach for accurate prediction of heme binding residues by a novel integrative sequence profile coupling position specific scoring matrices with heme specific physicochemical properties. In order to select the informative physicochemical properties, we design an intuitive feature selection scheme by combining a greedy strategy with correlation analysis.

**Results:**

Our integrative sequence profile approach for prediction of heme binding residues outperforms the conventional methods using amino acid and evolutionary information on the 5-fold cross validation and the independent tests.

**Conclusions:**

The novel feature of an integrative sequence profile achieves good performance using a reduced set of feature vector elements.

## Background

The heme-protein interactions are involved in a wide range of biological processes such as electron transfer, catalysis, signal transduction and control of gene expression [1]. To better understand the mechanism of heme-protein interactions and aid in heme related functional annotation, it is crucial to characterize and identify the binding sites of heme proteins[2]. It is well known that experimental techniques towards the determination of heme binding sites are prohibitively time-consuming and labour-intensive. Therefore, computational approaches are significantly in need for a rapid, high-throughput prediction of heme binding residues.

Recently, a pioneering method HemeBIND [3] is specifically designed to predict heme binding residues on heme proteins. At present, HemeBIND provides two complementary methods to distinguish heme binding sites from the rest of heme proteins. The main method integrates both sequence and structural features including evolutionary profile, solvent accessibility, depth, and protrusion index. Although the structural information can provide helpful insights for characterizing heme binding residues, there are currently only small fractions of 3D structures available for the heme proteins, which will limit the application scope of the structure-based methods. Therefore, HemeBIND also provides an alternative sequence-based method which can predict heme binding sites when only sequence information is available for heme proteins. The sequence-based classifier is constructed by the evolutionary information of amino acid sequences in the form of position specific scoring matrices (PSSM) that is generated by multiple sequence alignments.

In fact, in addition to PSSM, the physicochemical properties with high interpretability are also commonly used in the prediction of protein function from sequences[4-8]. Our previous work [9] also confirms the role of physicochemical properties in DNA-binding residues. However, to the best of our knowledge, no related work has incorporated physicochemical and biological properties from the Amino Acid Index (AAindex) database [10,11] to analyze and predict heme binding residues.

In the present study, we focus on the prediction of sequence-based heme binding sites, and attempt to integrate the physicochemical properties into PSSM, to provide additional insights to the heme binding residues and advance the prediction performance in actual applications. First, we use an intuitive feature selection scheme to choose an informative and compact subset of physicochemical descriptors in AAindex database. Then, we propose a novel integrative sequence profile, which is generated by coupling PSSM with the selected physicochemical properties. Evaluation experiments by using 5-fold cross validation on the training set and on the independent test demonstrate that our proposed approach outperforms the conventional methods based on PSSM profiles for prediction of heme binding residues.

## Methods

### Datasets

For training and testing, we used the datasets of heme proteins in previous studies [3,12]. The training set consists of 75 heme protein chains, derived from the nonredundant dataset of 89 heme proteins prepared by Fufezan et al [12]. After removing 14 chains whose HET group codes are not labelled as "HEM", we obtained the remaining 75 heme protein chains as the training set (PHeme-75). Since the heme proteins in PHeme-75 were constructed before March 2007, we used the other heme proteins collected after March 2007 as the testing set (PHeme-72) [3]. PHeme-72 is a nonredundant set of 72 heme protein chains, sharing no more than 30% sequence identity with any one of the 75 chains in the training set.

Following previous studies[3,13-16], we used the Ligand Protein Contact server to assign heme binding and nonbinding residues for the protein chains in the datasets. Among the training set of PHeme-75, we obtained 18584 residues with atomic coordinates, of which about 13.5% are heme binding sites. On the testing set of Pheme-72, there are 18581 residues, of which about 14.3% are heme binding residues.

### Feature construction

To build a classifier that can identify heme-binding residues from protein sequences, we constructed an effective strategy for integrating various features based on evolutionary profiles and physicochemical properties. For each target (or central) residue, the feature vector was constructed by the sliding window on a consecutive sequence for including the environmental information. In our study, we set *w *= 17 as the optimal size for building the sliding window (see details in Results section).

### Evolutionary information

This was obtained as the PSSMs generated by three iterations of PSI-BLAST [17] searches against NCBI nonredundant database (ftp://ftp.ncbi.nlm.nih.gov/blast/db/). The log odds values of 20 amino acid substitutions at a given alignment position were utilized to represent the evolutionary profile of a residue. The PSSM values were scaled to the range 0[1] by a standard logistic function [18]. One additional bit is utilized to deal with the terminal spanning windows. For the window size *w*, a vector of size (20+1)* *w *is used for representing a sample.

### Physicochemical property

Physicochemical properties (PP) are the most intuitive features for biochemical reactions and are widely applied in bioinformatics studies [4]. AAindex (http://www.genome.ad.jp/aaindex/) database is the collection of numerical indices representing various physicochemical properties of amino acids. The AAindex1 section of the AAindex database currently contains 544 amino acid indices. We removed the indices with missing values in AAindex1, with 531 entries left for use in our study. The raw values of these physicochemical properties were normalized to zero mean and unit standard deviation according to:

(1) $$P_{ij}^{\prime }\;=\;\frac{P_{ij}\;-\;\mu }{\sigma }$$

(2) $$\mu =\;\frac{1}{20}\;\overset{20}{\underset{j=1}{\sum }}P_{ij}$$

(3) $$\sigma \;=\;\sqrt{\frac{1}{20}\;\overset{20}{\underset{j=1}{\sum }}{\left(P_{ij}\;-\;\mu \right)}^{2}}$$

where *P_ij _*is the raw value of the *i*-th physicochemical property for the *j*-th amino acid type.

In order to select a subset of informative physicochemical properties, we first measured and ranked the predictive power of these 531 individual indices for correctly classifying all residues with atomic coordinates in Pheme-75 dataset using the area under the receiver operating characteristic curve (*AUC*). At this stage, no classifier is built so that no cross-validation scheme is required to calculate the *AUC *scores [19]. The *AUC *for an amino acid type is calculated in the same way as it would be for a classifier output. Each sample is associated with a feature value (e.g. an amino acid scale for a physicochemical property) and a positive or negative class label. A sample set is therefore converted into a sequence of feature values with an associated positive or negative label. The receiver operating characteristic curve and its *AUC *is calculated over this sequence.

**Figure 1:**
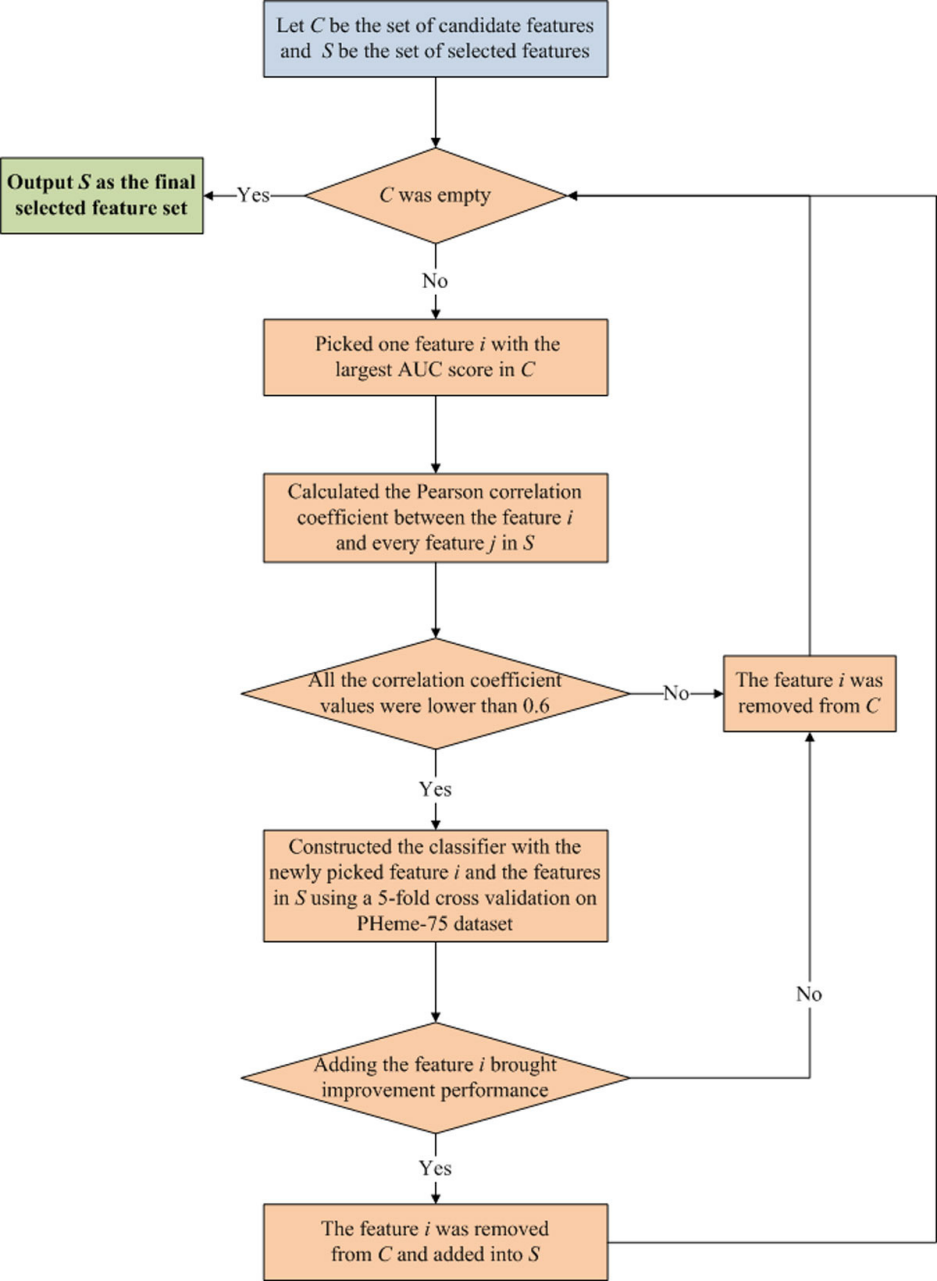
**Workflow of the proposed iterative feature selection process**.

We then designed a greedy approach in combination with correlation analysis for feature selection by constructing and assessing a series of heme binding sites predictors using 5-fold cross validation on the Pheme-75 dataset. Figure 1 shows the workflow of the iterative feature selection process. In this implementation, let *C *be the set of candidate features to be selected, and *S *be the set of features already selected. Initially, *C *was composed of the preselected features via *AUC *scores and *S *was empty. The features from *C *were then iteratively selected into *S *until *C *was empty. At the end, the features in *S *were used as the final feature subset in the whole feature selection process.

### Integrative sequence profile

**Figure 2:**
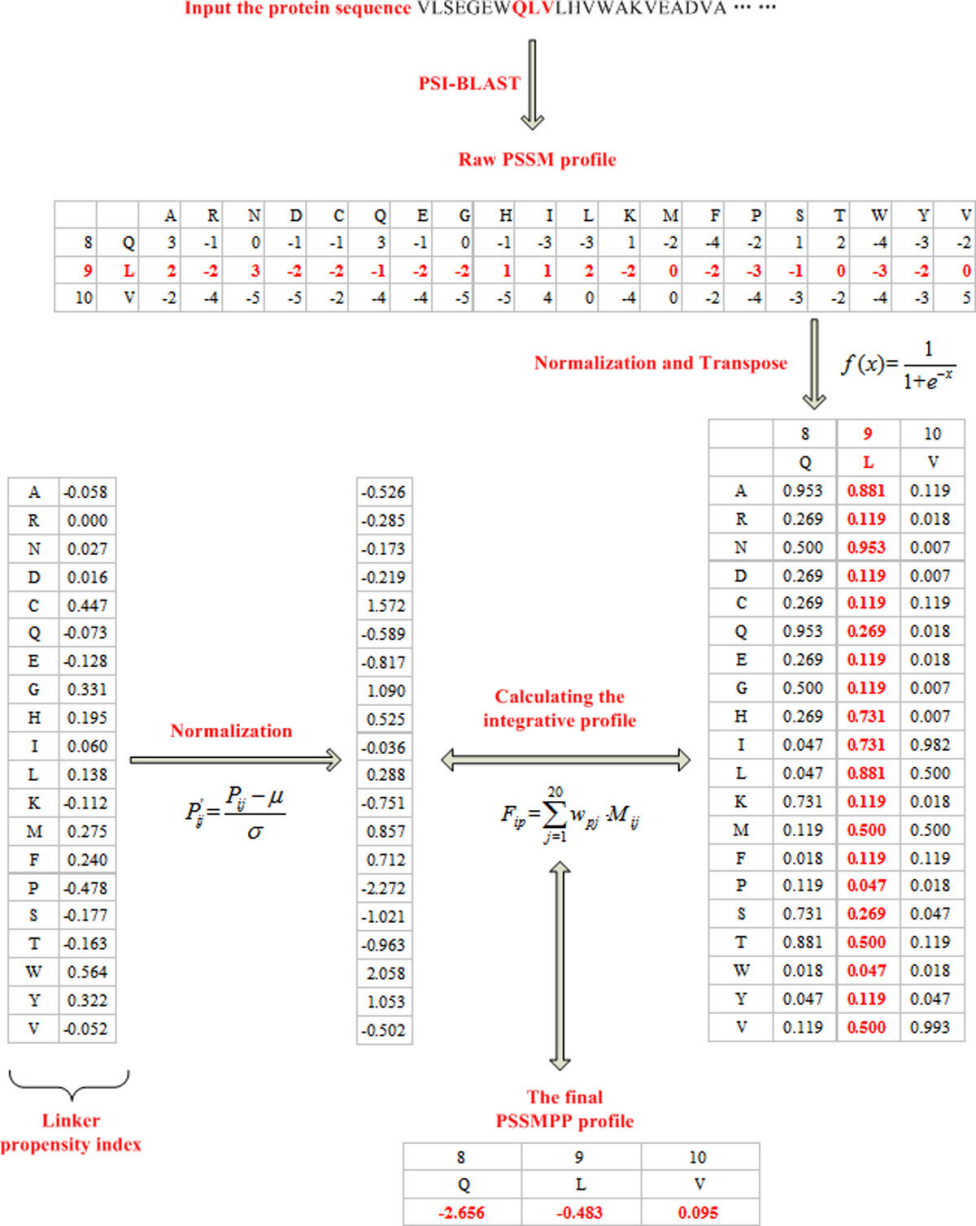
**Flowchart of generating the PSSMPP profile**. Given a heme binding protein sequence (PDB id: 1A6M; Chain: A), a window size of 3 is set for a simple illustration. The central residue is 9 L (residue number in the sequence; residue name), with its two neighbouring residues on both sides (8 Q and 10 V).

For the purpose of combining the predictive power of PSSM and PP, it is conventional to concatenate the vector elements of the PSSM and PP into a longer feature vector. Instead of using a concatenated vector with a high dimension, we implemented a condensed integrative profile by coupling PSSM with PP (called PSSMPP). The feature set of PSSMPP was constructed by summing up the 20 amino acid columns of the PSSM, weighted by the corresponding 20 amino acid values for a certain physicochemical property. Figure 2 presents an example for generating the PSSMPP profile vector. In the PSSMPP, the entry *F_ip _*of row *i *in the PSSM for a considering physicochemical property *p *is defined as follows, in much the similar way as previous work [20-22].

(4) $$F_{ip}\;=\;\overset{20}{\underset{j=1}{\sum }}w_{pj}\;\cdot M_{ij}$$

where

(1) *i *is the index of a position in the protein sequence;

(2) *w_pj_*is the normalized value of physicochemical property *p *for the *j*-th amino acid;

(3) *M_ij _*is the scaled value of the *j*-th type of amino acid in the position *i *of the PSSM.

### Model construction and evaluation

Support Vector Machines (SVMs) were applied to prediction of heme binding sites in our experiments. The SVMs are based on a rigorous statistical learning theory and have high generalization ability [23]. The SVM algorithms have demonstrated powerful performance in similar bioinformatics studies [24-27]. In this study, the SVM models were implemented with the radial basis function as a kernel using the *e1071 *library in R (http://cran.r-project.org/web/packages/e1071/), which provides the interface to the LibSVM. The models were first evaluated by 5-fold cross validation on the training set. Each classifier was trained using a data set comprising all positive samples from the cross validation fold and an equal number of randomly chosen negative samples. The models were further evaluated by the independent test on PHeme-72.

The performance of classification algorithms can be assessed by these metrics: accuracy (*ACC*), sensitivity (*SN*, also called recall), specificity (*SP*), precision (*PR*), Matthew's correlation coefficient (*MCC*) and F-measure (*F_1_*). These metrics are calculated using the numbers of true positives (*TP*), false positives (*FP*), true negatives (*TN*) and false negatives (*FN*) for each classifier. The performance measures are defined by the following equations.

(5) $$ACC=\;\frac{TP\;+\;TN}{TP\;+\;TN\;+\;FP\;+\;FN}$$

(6) $$SN\;=\;\frac{TP}{TP\;+\;FN}$$

(7) $$SP\;=\;\frac{TN}{TN\;+\;FP}$$

(8) $$\text{PR}\;=\;\frac{TP}{TP\;+\;FP}$$

(9) $$MCC\;=\;\frac{TP\;\times \;TN\;-\;FP\;\times \;FN}{\sqrt{\left(TP\;+\;FN\right)\;\times \;\left(TP\;+\;FP\right)\;\times \;\left(TN\;+\;FP\right)\;\times \;\left(TN\;+\;FN\right)}}$$

(10) $${\text{F}}_{1}\;=\;\frac{2\;\times \;SN\;\times \;\text{P}\;R}{\text{S}N\;+\;\text{P}\;R}$$

The receiver operating characteristic curve is a plot of the sensitivity versus (1-specificity) for a binary classifier at varying thresholds. The *AUC *was used as a main measure of classification performance throughout our work.

## Results

### Analysis of the selected physicochemical properties

Although some of the individual amino acid indices show modest discrimination abilities for distinguishing binding from nonbinding residues, the inter-feature redundancy makes it fail to improve the classification performance when they are combined together (data not shown). To rectify this problem, we performed a greedy feature selection approach in combination with correlation analysis to reach an optimal subset of features. Following the proposed iterative feature selection process, we obtained a subset of four physicochemical properties, which are listed in Table 1.

**Table 1:** The list of the selected subset of physicochemical properties on Pheme-75 dataset

ID	Description	AUC
QIAN880117	Weights for beta-sheet at the window position of -3	0.598
AURR980103	Normalized positional residue frequency at helix termini N"	0.593
AURR980118	Normalized positional residue frequency at helix termini C"	0.583
SUYM030101	Linker propensity index	0.573

From Table 2, most of the correlation coefficients among them were sufficiently low, which can partly justify using them in combination. These derived 4 physicochemical properties are related to alpha propensity[28], beta propensity[29] and the preference for linker regions of amino acids[30]. For example, when we use the amino acid scale SUYM030101 for analysis of heme binding and nonbinding residues, the heme binding residues are more abundant in the types of amino acids with high propensity in linker regions.

**Table 2:** The correlation coefficients among the four physicochemical properties on Pheme-75 dataset

	QIAN880117	AURR980103	AURR980118	SUYM030101
**QIAN880117**	-	-	-	-
**AURR980103**	0.020	-	-	-
**AURR980118**	-0.053	0.557	-	-
**SUYM030101**	0.107	0.104	0.286	-

### Prediction performance on Pheme-75 dataset using various feature sets

**Figure 3:**
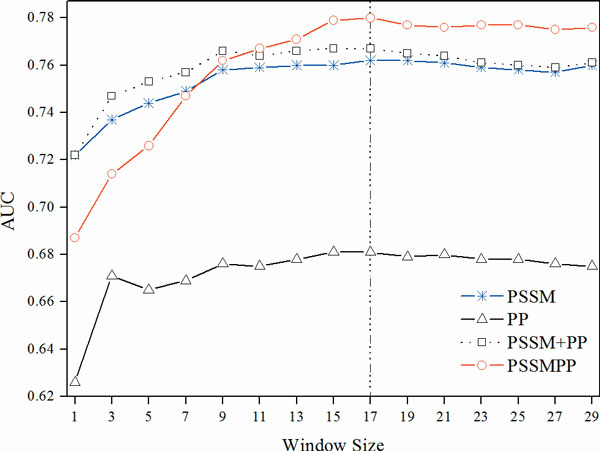
**Performance comparison of different features using 5-fold cross validation on Pheme-75 dataset at varying window sizes**.

In this section, we evaluated the classification performance of different feature sets using 5-fold cross validation on Pheme-75. Since the sliding window strategy was used to include the environment information, we should try different window sizes in order to find out an optimal window length. Figure 3 shows the performance of various features at varying window size from 1 to 29. In this study we adopted the window size of 17 unless otherwise stated, since all features achieved highest *AUC *values at this size. A closer examination of Figure 3 reveals that the conventional representation method of concatenating the vector elements of the PSSM and PP (PSSM +PP) yields marginally higher performance than PSSM at the cost of training time. However, the integrative profile of PSSMPP consistently outperforms all other feature sets when the window size is larger than 9. When using the optimal window size of 17, it is more obviously shown that PSSMPP performs better than the PSSM and the concatenated combination of PSSM with PP (PSSM +PP).

Table 3 presents the detailed metrics of the SVM classifiers using various features at the window size of 17. It is worth mentioning that the PSSMPP feature set improves precision and other metrics at the expense of the recall, but without dramatically compromising the recall measure. The moderate improvement of the overall performance is promising, considering the fact that PSSMPP used a significantly lower size of 85 ((4+1) *17) dimensions in the input vectors than the sizes of 357 and 425 ((20+4+1) *17), for PSSM and (PSSM+PP), respectively. The result is in agreement with the finding of the previous work [31] that a simple representation of the feature space could be much more powerful and efficient than the original data with all information included.

**Table 3:** Performance of different features on PHeme-75 dataset using 5-fold cross validation

Feature	ACC(%)	SN (%)	SP (%)	PR(%)	MCC	F_1_	AUC
PSSM	66.3	**73.4**	65.3	25.4	0.272	0.374	0.762
PP	62.9	63.4	62.7	21.4	0.184	0.319	0.681
PSSM+PP	67.8	72.1	67.2	26.2	0.279	0.381	0.767
PSSMPP	**71.1**	71.0	**71.1**	**28.5**	**0.306**	**0.403**	**0.780**

### Independent test on PHeme-72 dataset and comparison with HemeBIND

A true test of any prediction approach is to make predictions for the unseen dataset not utilized in training. In the section, we evaluated the prediction performance on an independent set of PHeme-72 using the best model trained on Pheme-75. As shown in Table 4, the testing performance of our model is not worse but even better than the training performance when using the same feature sets. The result suggests that the features we used here are not overfitting to the training set. In fact, for fairly comparing the predictive power of different features, we trained the SVM models using default parameters, resulting in the fact that the performance of the independent test set is even higher than that of the training set.

**Table 4:** Performance comparison of different methods on the independent test set of PHeme-72

Feature	ACC(%)	SN (%)	SP (%)	PR(%)	MCC	F_1_	AUC
Binary	67.9	61.8	68.9	24.9	0.225	0.355	0.718
PSSM	65.9	**75.2**	64.3	26.0	0.280	0.386	0.768
PSSMPP	**71.7**	71.6	**71.8**	**29.7**	**0.319**	**0.420**	**0.790**

In HemeBIND [3], two sequence-based classifiers were built by using amino acid binary pattern and PSSM, respectively. The authors have shown that the classifier based on PSSM significantly improved the prediction performance of the method based on unique representations (or binary encoding) of amino acid sequence and its environment. We implemented these two models, and compared our PSSMPP method with them using the same training and testing set, and the same definition of heme binding residues. Table 4 shows that our PSSMPP approach performs better than the binary and PSSM methods. Both in our work and Liu and Hu's study[3], the PSSM method outperforms the binary method.

## Conclusions

The main goal of the current study is to provide valuable insights to the heme binding residues and improve the classification performance based on heme protein sequences. In order to mine the informative physicochemical descriptors for heme binding residues, we designed a greedy approach in combination with correlation analysis for feature selection. Based on the selected physicochemical properties, we implemented an integrative sequence profile by coupling PSSM with four heme related physicochemical properties. The novel feature of PSSMPP achieves good performance using a reduced set of feature vector elements, whose size is significantly smaller than that of the conventional feature sets (i.e., PSSM). We believed that the reduced set of an integrative sequence profile feature can potentially be expanded to predict other functional residues on proteins.

## List of abbreviations used

PSSM: Position Specific Scoring Matrices; AAindex: Amino Acid Index; PP: Physicochemical Property; AUC: Area Under Curve; SVM: Support Vector Machine; ACC: Accuracy; SN: Sensitivity; SP: Specificity; PR: Precision; MCC: Matthew's Correlation Coefficient; F_l_: F-measure; TP: True Positive; FP: False Positive; TN: True Negative; FN: False Negative.

## Competing interests

The authors declare that they have no competing interests.

## Authors' contributions

YX and JL conceived and designed the experiments. YX implemented the prediction method. YX, WZ and TZ analyzed the data and wrote the manuscript. JL finalized the manuscript. All authors read and approved the final manuscript.
